# A Functional Comparison of the *3xP3* Promoter by Recombinase-Mediated Cassette Exchange in *Drosophila* and a Tephritid Fly, *Anastrepha suspensa*

**DOI:** 10.1534/g3.112.005488

**Published:** 2013-04-01

**Authors:** Marc F. Schetelig, Alfred M. Handler

**Affiliations:** U.S. Department of Agriculture, Agricultural Research Service, Center for Medical, Agricultural and Veterinary Entomology, Gainesville, Florida 32608

**Keywords:** site-specific recombination, genomic targeting, *Anastrepha suspensa*, insect pest management, *3xP3* promoter

## Abstract

Transposable elements are widely used as vectors for integrating transgenes into the genome of insects. However, the random nature of transposon vector integrations often results in mutations and makes transgene expression subject to variable genomic position effects. This makes reliable quantitative comparisons of different transgenes difficult and development of highly fit transgenic strains laborious. Tools for site-specific transgene targeting are essential for functional genomic comparisons and to develop the most advanced transgenic insect strains for applied use. Here we describe a recombinase-mediated cassette exchange gene targeting system based on Cre/*loxP* that is highly efficient in *Drosophila*, and for the first time in a non-drosophilid, the tephritid fly, *Anastrepha suspensa*. This system allowed a comparison of the *Drosophila* constitutive *polyubiquitin* promoter and the artificial *3xP3* tissue-specific promoter in the same genomic context within each species, showing that the widely used *3xP3* promoter is apparently nonfunctional in the tephritid fly.

Transposable elements have proven to be highly efficient vectors for the germline transformation of a variety of insect species. This began initially with use of the *P* element for fundamental studies in *Drosophila melanogaster* and more than a decade later, for both basic and applied studies in economically and medically important species using the *Hermes*, *Minos*, *mariner*, and *piggyBac* elements (Handler 2002). Despite the routine use of these vector systems in five orders of insects, there are significant drawbacks to the use of transposon-based vectors due to the random nature of their genomic integration (Schetelig *et al.* 2009b; Trauner *et al.* 2009). Insertional mutations within coding and regulatory regions can disrupt vital gene functions that are highly useful for genomic functional analysis but can be detrimental to fitness and viability in transgenic strains created for the control of pest populations or to beneficial insects used as predators or bioreactors. Most integrations are also subject to site-specific genomic position effects that can diminish or alter transgene expression, making functional comparisons unreliable, and the creation of strains having optimal transgene expression for applied use unnecessarily difficult (Horn *et al.* 2000; Schetelig *et al.* 2009a).

Strategies to address these issues have been developed for *Drosophila* using the site-specific recombination systems phiC31/*attP*, Cre/*loxP*, and FLP/*FRT* for genomic targeting (Groth *et al.* 2004; Horn and Handler 2005; Oberstein *et al.* 2005; Venken *et al.* 2006; Gao *et al.* 2008). For all systems, target-acceptor recombination sites were first integrated genomically using a transposon vector and were then targeted for recombination with reciprocal recombination site(s) within donor plasmids coinjected with a recombinase or integrase helper. Of these, only the integrase phiC31 has been used to insert *attB*-containing plasmids into *attP* landing sites in non-drosophilid genomes, including two *Aedes* species (Nimmo *et al.* 2006; Labbé *et al.* 2010) and the Mediterranean fruit fly, *Ceratitis capitata* (Schetelig *et al.* 2009b). Although the phiC31 system provided the first targeting system for non-drosophilids, it is limited by its unidirectional integration of the entire donor plasmid, typically including bacterial sequences as well as antibiotic resistance genes, and the potential for insertions into pseudo-*attP* sites within the genome (Groth *et al.* 2004). The FLP/*FRT* and Cre/*loxP* systems, however, have been used for recombinase-mediated cassette exchange (RMCE) in *D. melanogaster*, which is not restricted by these limitations (Horn and Handler 2005; Oberstein *et al.* 2005; Wimmer 2005). RMCE is based upon double recombination events, mediated by a recombinase, between two small heterospecific recombination sites within a genomic target site and a plasmid donor sequence. Depending on the number and position of independent recombination sites, RMCE allows for multiple insertion/deletion events of specific sequences at a single locus. In this fashion, transgene cassettes can be compared functionally in the same genomic context, and repetitively modified by the sequential deletion and addition of sequences.

The FLP/*FRT* recombinase system from the two-micron plasmid of yeast (Andrews *et al.* 1986) and the Cre/*loxP* system from bacteriophage (Siegal and Hartl 1996) both have 34-bp recombination sites consisting of two 13-bp inverted flanking repeats, separated by an 8-bp core, that specifically recombine with one another in the presence of FLP or Cre recombinase, respectively. Mutations in the 8-bp core create heterospecific sites, which are incompatible because only identical sites can recombine. Both *FRT* and *loxP* systems exhibited RMCE at relatively high frequencies in *Drosophila*, observed by the exchange of markers, although, unexpectedly, single insertional recombination also was observed (Horn and Handler 2005; Oberstein *et al.* 2005). A combination of these two systems for RMCE has also been successfully tested in cell culture that expands the possibilities for modification of any single locus (Anderson *et al.* 2012). RMCE in non-drosophilids would be an important technique, especially to generate transgenic strains for insect population control, using target sites known to be nonsusceptible to mutational and position effects. The ability to improve such strains by gene replacement and addition would be a highly efficient alternative to creating completely new strains by transposon-mediated transformations that would require extensive evaluation for strain fitness and transgene expression for risk assessment evaluation. Importantly, transposon vectors used to create optimal target sites could be efficiently stabilized by a postintegration immobilization process providing enhanced environmental safety for transgenic strains created for field release applications (Handler *et al.* 2004; Schetelig *et al.* 2009b).

In support of this goal, we describe an RMCE system for a non-drosophilid species, the Caribbean fruit fly, *Anastrepha suspensa*, using the heterospecific *loxN* and *lox2272* recombination sites (Araki *et al.* 2002; Livet *et al.* 2007) with a *D. melanogaster hsp70*-regulated Cre-recombinase. The efficacy of this system was tested in a comparative functional analysis of the *3xP3* artificial promoter, proving that this widely used promoter derived from the highly conserved *Pax-6*/*eyeless* system is, thus far, uniquely non-functional in a tephritid species.

## Materials and Methods

### Insect rearing

An inbred wild-type (WT) colony of *A. suspensa* (Homestead, FL) and the *Drosophila melanogaster white*^118^ (*w*^118^) mutant strain were maintained at 25° and reared under standard laboratory conditions (Saul 1982; Roberts 1986). All embryonic, larval, and pupal stages of *A. suspensa* were reared at 27° and 60% humidity on a 12-hr light:12-hr dark cycle.

### Cloning

The vector *pBXLII_PUbEGFP_TREhs43-CctraI-Alhid^Ala2^_loxN-3xP3-FRT-AmCyan_lox2272_loxP_attP235* (*TRE-CctraI-Alhid^Ala2^*; #443) was described previously (Schetelig and Handler 2012b). The Cre-helper plasmid *phsp70-Cre* (#445) was generated by recombining three fragments using the GeneArt Seamless Cloning Kit (Invitrogen) as follows: (1) a 3.7-kb *Eco*RV-digested fragment of *phsp-pBac* (Handler and Harrell 1999), containing the *hsp70* promoter; (2) a 0.8-kb polymerase chain reaction (PCR) fragment of *Cre*, isolated with primer pair P815-P816 on a *Cre*-containing plasmid generously provided by Dr. J. Livet (Inserm); and (3) a 0.6-kb PCR fragment of the *piggyBac* 3′-UTR, isolated using primer pair P817-P818 on *phsp-pBac*. The Platinum Taq Polymerase was used for PCRs with the following conditions: 1 min at 95°; 30 cycles of 15 sec at 94°, 30 sec at 55°, 1 min at 72°; and 2 min at 72°.

The construct pSL_loxN-PUbDsRed-lox2272 was generated by ligating the 3.7-kb pSL-loxN-lox2272 *Hpa*I-*Sma*I digested fragment from M879 and the 2.9-kb PUbDsRed.T3 *Hpa*I-*Sma*I-digested fragment from #1425 (Schetelig and Handler 2012a). M879 was created by ligating the *Xho*I-digested fragment loxN-3xP3FRTAmCyan-lox2272 from M746 (Schetelig and Handler 2012b) into the *Xho*I-*Sal*I cut pSLfa1180fa (Horn and Wimmer 2000).

### Germline transformation

Germline transformation experiments were performed by microinjection of the *piggyBac* target site construct #443 (500 ng/µL) with the *phsp-pBac* transposase helper plasmid (200 ng/µL) into WT *Drosophila* embryos as described previously (Handler and Harrell 1999; Handler 2000). G1 offspring were selected by enhanced green fluorescent protein (EGFP) epifluorescence using a Leica MZ FLIII microscope and a YFP filter set (ex: 500/20; em: 535/30). Independent homozygous strains were established by single pair inbreeding for successive generations with testing by segregation analysis of transformants outcrossed to WT flies. Transgenic *A. suspensa* lines carrying the #443 *piggyBac* cassette were generated and described earlier as a lethal effector construct (Schetelig and Handler 2012b).

### Recombinase-mediated cassette exchange

Cre-RMCE was achieved by transformation, in which the RMCE donor plasmid *pSL_loxN-PUbDsRed-lox2272* (250 ng/µL) and the helper plasmid *phsp70-Cre* (150 ng/µL) were coinjected into RMCE target line embryos, without subsequent heat shock. Male or female G_0_ adult survivors (*D. melanogaster* or *A. suspensa*) were mated individually to three virgin *w*^-^ females or males (*D. melanogaster*) and WT virgin females or males (*A. suspensa*), respectively. Their progeny were screened for the presence of eye and body markers by epifluorescence microscopy. Three subsequent backcrosses of transgenic males or females to WT females or males were first performed to verify a transgenic/WT progeny ratio of 1:1, and the same fluorescent marker tissue specificity consistent with single vector integrations. Independent homozygous strains were then established by single pair inbreeding for successive generations with testing by segregation analysis of transformants outcrossed to WT flies. Three filter sets (Leica) were used for fluorescent marker detection: TxRed for DsRed (ex: 560/40; em: 610 LP), CFP for AmCyan (ex: 436/20; em: 480/40), and YFP for EGFP (ex: 500/20; em: 535/30).

### Verification of RMCE and expression analysis

RMCE was verified by isolating the complete *loxN*-Marker*-lox2272* cassette from genomic DNA by PCR using primer pair P898/P899 (P898: ACGGGAAGTATCAGCTCGACCATGG; P899: GAGCGCGACTTGTACAGCCATGG). Fragments were subcloned into the pCR4 vector (Life Technologies) and sequenced (Macrogen). All primers were designed using Geneious 5.6 software (Biomatters). Total RNA was isolated from adult heads using TRIreagent (Molecular Research Center) with 1 µg of total RNA used for cDNA synthesis with the iScriptTM cDNA synthesis kit (BioRad). For both genomic DNA and adult head cDNA, PCRs were performed targeting the AmCyan (primer pair P915: TCCACACCTCCTACAAGACCAAG / P916: GGTCAGCTGCACGCTGTTGC) and EGFP (primer pair P913: CAGAACACCCCCATCGGCGACGGC / P914: TACTTGTACAGCTCGTCCATG) sequences.

Real-time quantitative PCR (qPCR) was performed on ~100 ng of cDNA, quantified on a NanoDrop 2000 (Thermo Scientific), using the iQ SYBR Green Supermix in a Chromo4 real-time PCR detector (BioRad). PCR cycling conditions were: 95° for 5 min; 45 cycles of 95° for 15 sec, 60° for 10 sec, and 72° for 10 sec with a plate read at the end of each cycle. All reactions were performed on three biological replicates. Gene specific primers for *AmCyan* (P915/P916) and *EGFP* (P913/P914) were used.

Amplified products from randomly selected samples were analyzed on a 2% agarose gel, subsequently cloned into pCR4-TOPO vector, and sequenced to confirm specificity of the *AmCyan* and *EGFP* amplifications. For absolute quantification, the *AmCyan* transcript levels were normalized against *EGFP* and compared with a standard curve against the AmCyan-carrying #443 plasmid. Calculations were performed using the Opticon Monitor 3 software (BioRad) and the standard curve was prepared on the 12,576-bp long plasmid #443 using five dilutions (300,000; 30,000; 3000; 300; 30 copies) according to Applied Biosystems (http://www3.appliedbiosystems.com/cms/groups/mcb_marketing/documents/generaldocuments/cms_042486.pdf).

## Results

### Validation of markers and Cre/*lox* vector and helper plasmids in *D. melanogaster*

**Figure 1:**
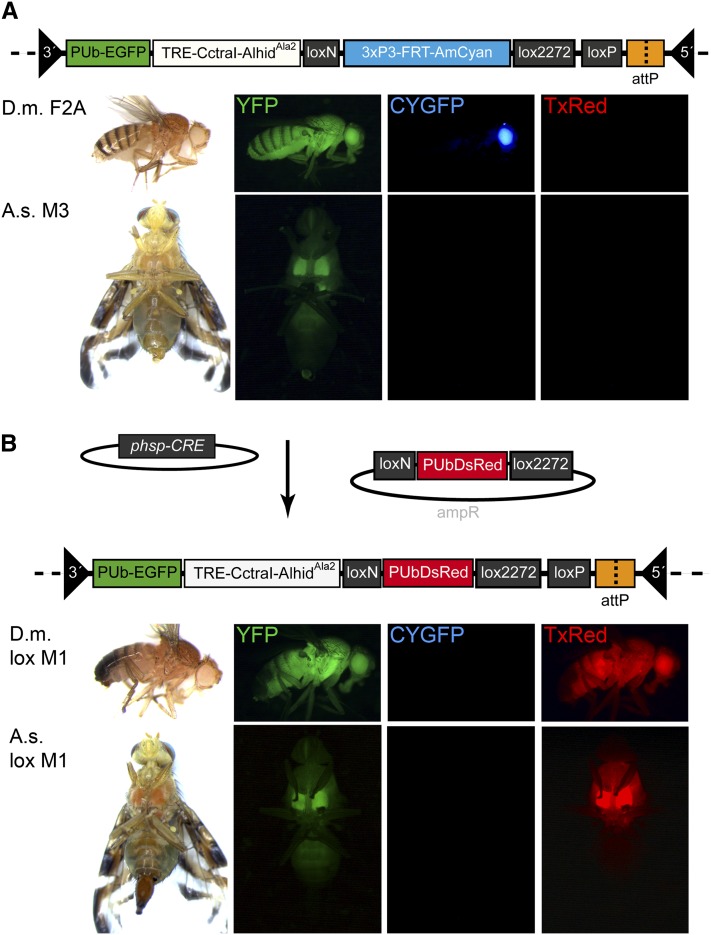
Recombination-mediated cassette exchange. A schematic of the *piggyBac* target site vector is shown on top, with images of the *Drosophila* (*D.m*.) and *Anastrepha* (*A.s*.) transformant strains below, carrying a green fluorescent body marker and an eye-specific blue fluorescent marker (A). A schematic of the target site after successful RMCE is shown, where the blue fluorescent marker is exchanged for a red body marker (B). All flies were observed under brightfield conditions (left) and epifluorescent microscopy with the filter sets YFP, CYGFP, and TxRed (right).

Before Cre-mediated cassette exchange in a non-drosophilid insect was tested, all components were functionally verified in *D. melanogaster*, for which *lox*-site RMCE was previously demonstrated, but with the use of a *P* vector and markers specific to that species (Oberstein *et al.* 2005). A *piggyBac* target-site transformation vector, *pBXLII_PUbEGFP_TREhs43-CctraI-Alhid^Ala2^_loxN-3xP3-FRT-AmCyan_lox2272_loxP_attP235* (#443), carrying the *3xP3-AmCyan* eye marker flanked by the heterospecific *lox* sites *loxN* and *lox2272* (Livet *et al.* 2007), together with the fluorescent marker *PUb-nls-EGFP* (Handler and Harrell 1999), was used for germline transformation of *D. melanogaster w^−^* flies (Figure 1A). Of 220 eggs injected, 18 adults survived that were backcrossed to *w^−^* males or virgin females. Sixteen crosses were fertile yielding two transgenic lines, Dm-F2A and Dm-M8A, that were identified by screening for both blue fluorescent eyes/ocelli (*3xP3-AmCyan* marker) and green fluorescent adult body tissue (*PUb-nls-EGFP* marker), using epifluorescence microscopy (Figure 1). The two independent lines were separately inbred to homozygosity.

**Figure 2:**
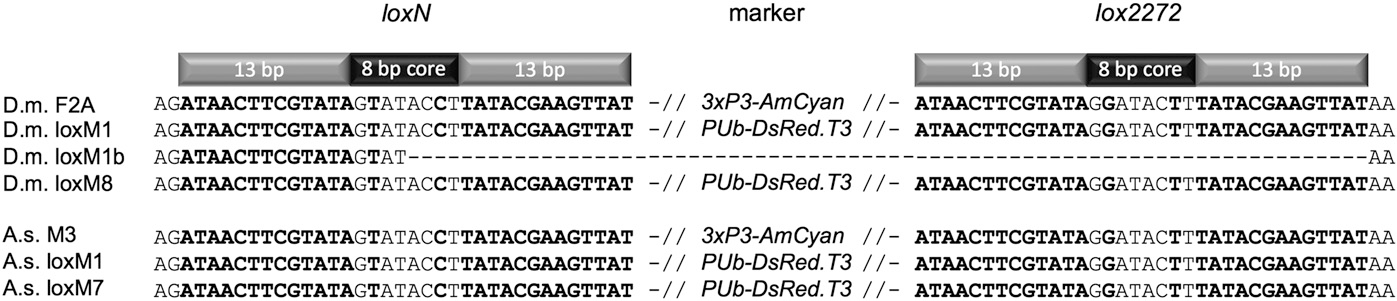
RMCE in *D. melanogaster* and *A. suspensa*. A comparison of sequenced PCR amplicons of the original strains carrying the *3xP3-AmCyan* marker and the subsequent RMCE strains carrying the *PUb-DsRed.T3* marker between heterospecific *loxN* and *lox2272* sites show the precise RMCE. The imprecise excision in the line Dm-loxM1b is shown.

To catalyze *lox* site recombination, the helper plasmid *phsp-Cre* was generated having the *Cre* recombinase gene under *Dmhsp70* promoter control. The helper was coinjected with the donor vector, *pSL_loxN-PUbDsRed-lox2272*, into homozygous Dm-F2A preblastoderm embryos. A total of 109 embryos were injected, from which 17 adults emerged that were single-pair mated to *w^−^* males or virgin females. Eleven matings were sterile, whereas six matings led to viable offspring. In two of these six crosses (Dm-loxM1, Dm-loxM8), successful cassette exchange was observed by loss of the blue fluorescent eye/ocelli marker expression that was replaced by whole-body expression of DsRed (Figure 1B). In addition to precise RMCE of the markers, a third line (Dm-loxM1b) showed only loss of the eye marker. To verify molecularly both successful RMCE and eye marker excision, the complete marker cassettes flanked by the heterospecific *lox*-sites were isolated from genomic DNA of both lines and sequenced. The obtained sequences for Dm-loxM1 and Dm-loxM8 indicated precise heterospecific cassette exchange involving the *loxN* and *lox2272* sites, whereas Dm-loxM1b showed a loss of the marker cassette by imprecise excision (Figure 2).

### Cre/*lox* RMCE in *A. suspensa*

Five transgenic *A. suspensa* lines carrying the *piggyBac* vector, #443 were generated previously (Schetelig and Handler 2012b), and, interestingly, *3xP3*-driven AmCyan could not be detected in any of the lines whereas the *PUb*-driven EGFP marker was clearly visible. RMCE was tested in the As-M3 line by injecting the *phsp-Cre* helper and the *pSL_loxN-PUbDsRed-lox2272* donor vector into dechorionated preblastoderm embryos. A total of 230 embryos were injected in two independent experiments (#1: 115; #2: 115), from which 12 adults (#1: 5; #2: 7) emerged that were single-pair mated to *A. suspensa* WT males or virgin females. Four matings (#1: 1; #2: 3) were sterile whereas eight matings led to viable offspring. In two (#1: 1; #2: 1) of the eight crosses (As-loxM1, As-loxM7) the additional expression of DsRed in whole flies indicated successful RMCE (Figure 1). The precise cassette exchange in both lines was then verified by amplifying and sequencing the complete *PUb-DsRed* marker cassette flanked by the heterospecific *lox*-sites (Figure 2).

### *3xP3* promoter analysis in *A. suspensa*

**Figure 3:**
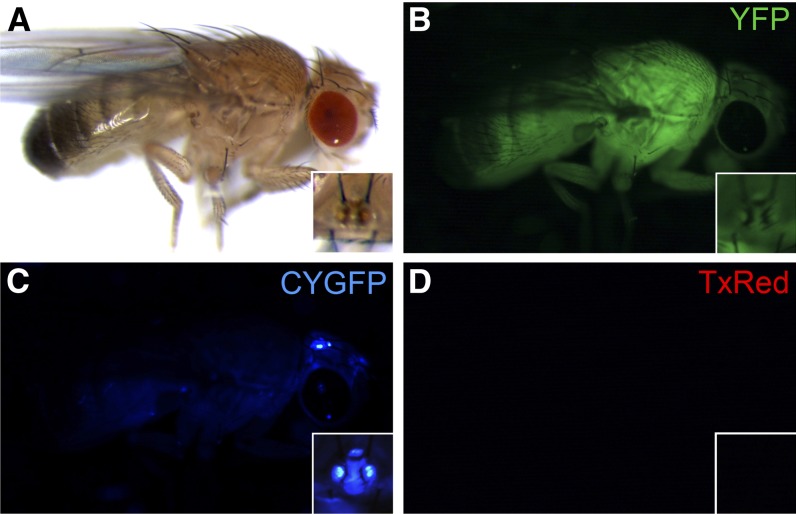
Epifluorescence from a Dm-F2A fly in a *white^+^* genetic background. The same fly was observed under brightfield conditions (A) and epifluorescent microscopy with the filter sets YFP (B), CYGFP (C), and TxRed (D). AmCyan fluorescence is highly reduced/quenched in the pigmented eye of Dm-F2A *w^+^* flies (C). Blue fluorescent ocelli and the pseudopupil indicate *3xP3*-marker function (B). Magnified images of the ocelli are inset at the bottom right of each whole-body image.

In addition to establishing RMCE in a tephritid, the construct #443 also allowed a determination of *3xP3* promoter function in *A. suspensa*, in comparison with *D. melanogaster*. The strategy was to use the double-marked construct to compare expression of the *3xP3*- and the *D. melanogaster polyubiquitin* (*PUb*)-regulated markers in the same genomic context within each species. As shown in the *Drosophila* and *Anastrepha* transgenic lines in Figure 1, the *PUb*-regulated markers are clearly visible in both species, whereas 3xP3-AmCyan expression is exclusively detected in ommatidia and ocelli of *Drosophila*, but in neither tissue in *A. suspensa*. One particular difference between the species is their eye pigmentation. *Drosophila* strains were generated from a white eye mutant strain, *w*^118^, whereas transgenic *Anastrepha* lines had red pigmented WT eyes. To determine whether pigmentation may have masked detection of 3xP3-regulated marker expression in *Anastrepha*, *Drosophila* strains were backcrossed to the WT Oregon-R strain. The resulting transgenic flies having red pigmented eyes, did show weak *AmCyan* expression in the pseudopupil and strong blue fluorescence in the ocelli (Figure 3) consistent with earlier studies (Berghammer *et al.* 1999). However, after exchanging *3xP3-AmCyan* for *PUb-DsRed* by RMCE, both species exhibited red fluorescence in the whole body (Figure 1B). Therefore the integration site and composition of the construct were not responsible for the absence of the blue fluorescence in the eyes of *A. suspensa*.

**Figure 4:**
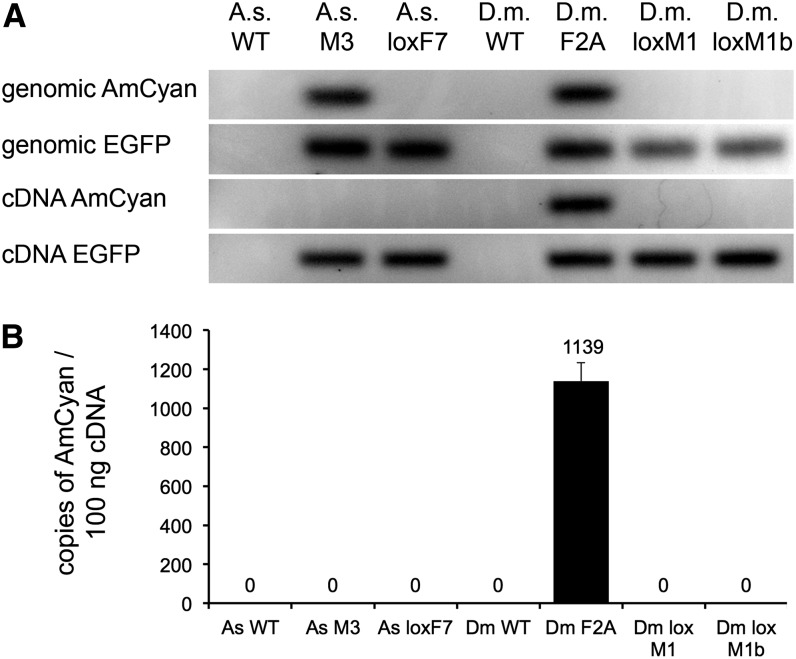
Qualitative and quantitative verification of the markers. Detection of AmCyan and EGFP by PCR on genomic DNA and cDNA (A). Absolute quantitative PCR on AmCyan was used to measure the expression of AmCyan in transcript copies per 100 ng of cDNA (B).

The lines were then analyzed molecularly on two levels. First, PCR fragments of *AmCyan* and *EGFP* were isolated, subcloned, and sequenced to qualitatively reveal the absence or presence of the markers. This was performed on genomic and cDNA from the original lines as well as lines created by RMCE (Figure 4A). Amplicons from genomic DNA confirmed that the *AmCyan* marker gene was present in the lines As-M3 and Dm-F2A as expected. In contrast, transcripts could not be amplified from As-M3 cDNA but were amplified from Dm-F2A. As a control, the *EGFP* marker was amplified from all genomic and cDNA samples of transgenic *Anastrepha* and *Drosophila* flies, whereas no amplicons were detected in WT strains tested. This finding led us to presume that expression levels of *AmCyan* under *3xP3* artificial promoter control were highly reduced in *A. suspensa* and possibly nonexistent.

Absolute real-time qPCR was then used to investigate differences in transcript copy number from the *AmCyan* transgene in all transgenic lines (Figure 4B). No copies were detected in the line As-M3, which is consistent with the absence of blue fluorescence in the eyes (Figure 1A). The control line, As-loxF7, in which the *3xP3-AmCyan* was exchanged by RMCE for *PUb-DsRed*, and the *Anastrepha* WT strain did not express *AmCyan*. This clearly demonstrated that *AmCyan* is not expressed in *A. suspensa*. In the positive control *Drosophila* strain, Dm-F2A (visible blue eyes with epifluorescence microscopy), 1139 copies of *AmCyan* were detected and none in the lines Dm-loxM1, as well as the *D. melanogaster* WT line in which *3xP3-AmCyan* does not exist.

## Discussion

Here we have shown genomic targeting of recombinant DNA to a specific locus by RMCE between heterospecific *lox* sites in a non-drosophilid insect species. Compared with the phiC31/*attP* system, which has been established as a unidirectional targeting system in mosquitoes and a tephritid species, RMCE methodology is particularly advantageous as a means to not only target recombinant DNA constructs to a specific genomic site, but to subsequently modify the construct by sequence addition, deletion, or exchange. This significantly advances our ability to functionally characterize genes of interest and facilitates the creation and improvement of transgenic strains for applied use. A particular advantage is that cassette exchange events can be performed for comparative gene expression analysis in an identical genomic context to compare individual genes, or complex recombinant constructs, subject to varying genomic influences on transgene expression. The exchange efficiency of 25–33% in our small-scale experiments demonstrates that the mutant *lox2272* and *loxN* sites are well suited for RMCE in *A. suspensa* and *D. melanogaster*. Because each cassette exchange of a transgenic line should result in the same genomic modification, accomplishing RMCE reliably is, in general, more important than high efficiency alone, unlike germline transformation where several (if not many) different insertions are desired to select optimal transformant lines.

Differences between the system presented here and the first *lox* RMCE in *Drosophila* (Oberstein *et al.* 2005), beyond use of the *P* element vector and markers specific to *Drosophila*, include the source of Cre recombinase and the successful use of *loxN* in *A. suspensa* and *D. melanogaster*. Alterations between the *loxN*, *lox2272*, and the original *loxP* site are two mutations in the 8-bp core at positions two and seven (Livet *et al.* 2007), and we found RMCE using *loxN* to be strictly heterospecific in both species. The non-exchange loss of *3xP3-AmCyan* in the *Drosophila* line Dm-loxM1b is not understood, but was presumably caused by an imprecise excision or break between the *lox* sites.

In the process of achieving RMCE, the use of a helper plasmid having Cre recombinase, fused to the *Drosophila hsp70* promoter, proved to be more stable than capped recombinase mRNA that tended to degrade quickly and negatively affected the survival rate of injected embryos. The *hsp70-Cre* helper plasmid allowed for highly efficient RMCE, up to 33%, compared to 5–9% efficiency in *D. melanogaster* reported earlier using an *hsp70/Mos1*-regulated recombinase. Together, the results clearly show that DNA-based expression of Cre recombinase can be highly effective in catalyzing in-vivo RMCE using *loxN* and *lox2272* recombination sites in *A. suspensa*, that should be extended to other tephritid species, if not other insects, as well.

In the process of testing RMCE we compared the *3xP3* eye-specific promoter and the *polyubiquitin* (*PUb*) constitutive promoter in the same genomic context. The artificial *3xP3* promoter, originally tested in *D. melanogaster*, contains three binding sites for *Pax-6*/*eyeless* homodimers upstream to a TATA box. This is an evolutionarily highly conserved system that was described as the master regulator of eye development throughout the animal kingdom (Gehring 2002). This view is consistent with the very broad function of *3xP3* (Sheng *et al.* 1997; Berghammer *et al.* 1999) as a promoter for fluorescent protein genes, which have been successfully used as an adult eye and ocelli marker for transgenesis in *Drosophila*, the housefly, beetles, butterflies, mosquitoes, and even flatworms (Horn *et al.* 2000; Hediger *et al.* 2001; Kokoza *et al.* 2001; Gonzalez-Estevez *et al.* 2003; Lorenzen *et al.* 2003; Marcus *et al.* 2004). It also promotes expression in the larval nervous system, which has been highly useful in identifying silkmoth transformants (Thomas *et al.* 2002). Nevertheless, several attempts by us and other laboratories to use 3xP3-fluorescent protein markers in the tephritids *C. capitata*, *A. suspensa*, and *Anastrepha ludens* have failed to produce an identifiable transgenic phenotype (including *C. capitata white eye* host strains). After exchanging the nonfunctional *3xP3*-driven *AmCyan* cassette for a ubiquitously expressed *PUb*-driven *DsRed* marker by RMCE, we found that DsRed was visible by epifluorescence microscopy in the whole body of *A. suspensa*. Therefore, the lack of visible fluorescence from *3xP3-AmCyan* in *A. suspensa* was not related to a defect or genomic position effect because the same construct functioned in *Drosophila*, and an equally positioned *PUb* promoter expressed the red fluorescent protein unambiguously in both species. The lack of promoter function for *3xP3* in *Anastrepha* was further verified by qPCR that showed the absence of *AmCyan* transcripts whereas, in contrast, a high transcript level was obtained from the same *3xP3*-marker in the *Drosophila* lines.

The *3xP3* promoter consists of three P3 binding elements and a minimal promoter from the *Drosophila hsp70* gene. Cross-species expression differences due to this minimal promoter are unlikely because it has been used successfully in *A. suspensa* and another tephritid, *Ceratitis capitata* (Schetelig *et al.* 2009a; Schetelig and Handler 2012a). A modification of the original *3xP3*-regulated markers developed in *Drosophila* (Berghammer *et al.* 1999) is the insertion of an 84-bp linker, which includes an *FRT* recombination site, in between the promoter and the fluorescent protein gene. The modified *3xP3-FRT*-AmCyan construct was used in the RMCE vector, and although it clearly was functional in *Drosophila*, it has not been tested in species known to express *3xP3*-regulated markers lacking the FRT insert. Thus, the present data cannot formally exclude a negative effect of the inserted sequence on *3xP3* function in non-drosophilids.

To our knowledge this is the first report indicating that *3xP3* is nonfunctional in an insect species and possibly an insect family. Given its routine use for marker expression in lepidoptera, coleoptera, and several dipteran species, this is unexpected and not simple to explain. It certainly raises the possibility for differences in the highly conserved mechanism of eye development between tephritids and other insects, which might range from control by a distinct gene in *Anastrepha*, to a variation in binding sites for the P3/RSC1 elements. An in-depth analysis for this mechanism in tephritid species will be required, and RMCE methodology should play a pivotal role in the comparative studies necessary to elucidate our understanding of *eyeless* function.
